# Polaronic Conductivity in Iron Phosphate Glasses Containing B_2_O_3_

**DOI:** 10.3390/ma13112505

**Published:** 2020-05-30

**Authors:** Luka Pavić, Stjepko Fazinić, Hüseyin Ertap, Mevlüt Karabulut, Andrea Moguš-Milanković, Ana Šantić

**Affiliations:** 1 Ruđer Bošković Institute, 10000 Zagreb, Croatia; sfazinic@irb.hr (S.F.); mogus@irb.hr (A.M.-M.); 2 Department of Physics, Kafkas University, 36100 Kars, Turkey; huseyinertap@kafkas.edu.tr; 3 Department of Physics, Gebze Technical University, 41400 Gebze, Kocaeli, Turkey; mevlutk@gtu.edu.tr

**Keywords:** iron phosphate glass, polaronic conductivity, glass–former oxide, structure–property relationship, impedance spectroscopy

## Abstract

We report on the electrical properties of glasses with nominal composition *x*B_2_O_3_–(100 − *x*)[40Fe_2_O_3_–60P_2_O_5_],*x* = 2–20, mol.%. The conduction transport in these glasses is polaronic and shows a strong dependence on Fe_2_O_3_ content and polaron number density. The changes in DC conductivity are found not to be directly related to B_2_O_3_, however structural changes induced by its addition impact frequency-dependent conductivity. All glasses obey Summerfield and Sidebottom procedures of scaling conductivity spectra indicating that the polaronic mechanism does not change with temperature. An attempt to produce a super-master curve revealed that shape of the conductivity dispersion is the same for glasses with up to 15.0 mol.% B_2_O_3_ but differs for glass with the highest B_2_O_3_ content. This result could be related to the presence of borate units in the glass network. Moreover, the spatial extent of localized polaron motions increases with the decrease of polaron number density, however, this increase shows a larger slope than for previously reported iron phosphate glasses most probably due to the influence of B_2_O_3_ on glass structure and formation of polarons. While Summerfield scaling procedure fails, Sidebottom scaling yields a super-master curve, which indicates that polaronic hopping lengths also change with changing polaron number density in these glasses.

## 1. Introduction

Iron phosphate-based glasses belong to a family of electronically conducting materials in which the conduction mechanism follows the small polaron hopping theory [1,2,3,4,5,6,7]. The polaronic conductivity in these glasses originates from the electron transfer between ferric and ferrous ions and, consequently, shows a strong dependence on the total amount of iron oxide, fraction of ferrous/ferric ions and average distance between them. While literature reports numerous studies on the influence of these parameters on the polaronic conductivity [8,9,10], as well as the influence of various modifier oxides, mainly transition metal or alkali oxides which themselves are potential active electrical contributors [6,11,12,13,14,15,16,17,18], investigations of the impact of the additional glass-forming oxide on the polaronic transport in iron phosphate glasses are sparse.

In ionically conducting glasses containing a fixed amount of alkali oxide, a gradual substitution of one glass-forming oxide by another produces a pronounced conductivity maximum at the glass composition with the highest fraction of structural units from both glass-forming oxides [19,20,21,22,23]. This effect, called mixed glass-forming effect, has been identified in various combinations of glass-forming oxides, including those based on phosphorous pentoxide and clearly reflects the importance of the glass structure in ionic transport. Bearing in mind that the mechanism of polaronic transport is very different in nature to the ionic one, it is unrealistic to expect such a straightforward relationship between polaronic conductivity and changes in the mixed glass-forming network. However, polaronic conductivity of iron phosphate glasses does depend on the local structural surroundings of iron ions and their long-range connectivity in the glass network which is inherently related to the basic features of the glass network.

In this study, we report a detailed analysis of electrical properties of iron phosphate (IP) glasses of the molar composition 40Fe_2_O_3_-60P_2_O_5_ doped with boron oxide (IBP glasses). The structural properties of this glass system are relatively well examined [24,25,26]. It was shown that B_2_O_3_ enters the glass network as BO_4_ tetrahedral units and increases the chemical and thermal stability of the glass [24,25]. Moreover, in our previous study, we showed that the replacement of a small amount of Fe_2_O_3_ by B_2_O_3_ in glasses with fixed phosphate content (60 mol.%) induces local structural disorder which influences polaronic transport giving rise to a deviation from the Summerfield scaling of conductivity spectra [27].

The present work aims to investigate this interesting behavior further and to examine the structural-electrical properties relationship in glasses in which up to 17.7 mol.% of B_2_O_3_ is systematically added to 40Fe_2_O_3_-60P_2_O_5_ glasses at the expense of both Fe_2_O_3_ and P_2_O_5_. Such variation in composition in which Fe/P ratio is kept constant (0.67), involves smaller changes in overall Fe_2_O_3_ amount in comparison to the previously studied system [27], but different structural properties. Indeed, the structural study of both glass systems showed that the addition of B_2_O_3_ with simultaneous decrease of contents of both Fe_2_O_3_ and P_2_O_5_ decreases the thermal stability of glasses, whereas an opposite trend is observed for an exchange of just Fe_2_O_3_ by B_2_O_3_ [24]. Therefore, in this study, we present a detailed analysis of the electrical properties of these glasses in a wide range of temperatures and frequencies in order to address the role of glass structure in the processes of polaronic transport and to determine parameters that govern them.

## 2. Materials and Methods

The batch composition of the glasses selected for this investigation is *x*B_2_O_3_–(100 − *x*)[40Fe_2_O_3_–60P_2_O_5_], *x* = 2–20, mol.%. Glasses were prepared by melting homogenous mixtures of reagent grade chemicals (B_2_O_3_–Fe_2_O_3_–P_2_O_5_) (Sigma Aldrich, Darmstadt, Germany) in appropriate quantities in high-density alumina crucibles at 1150–1200 °C in the air for 1–2 h. The details of the preparation procedure are given in Ref [24]. Melt is quenched by pouring into a 1 cm × 1 cm × 5 cm steel mold. The resulting glass bars are then moved quickly to an annealing furnace, annealed at 475 °C for 3 h and slowly cooled down to room temperature. The glass samples are designated in accordance with the amount of boron oxide in batch composition. For instance, B-2 glass contains 2 mol.% of B_2_O_3_. The powder X-ray diffraction (XRD-6000 diffractometer, Shimadzu, Kyoto, Japan) studies are performed to confirm the amorphous nature of the samples [24]. The exact composition of glass samples was determined by particle induced gamma-ray emission (PIGE) technique [28] using 3 MeV protons from the Ruđer Bošković Institute (RBI) Tandetron accelerator (High Voltage Engineering Europa B.V., Amersfoort, The Netherlands) and high-resolution high purity germanium (HPGe) detector (Canberra Industries, Meriden, CT, USA) to detect gamma rays from the measured samples. Gamma rays of boron (429 and 718 keV), phosphorus (1266 keV), iron (847 keV) and oxygen (871 keV) were used for quantitative analysis of spectra and determination of the elemental composition of samples. Proton elastic backscattered spectra (EBS) and collected proton charges were recorded simultaneously to help in the analysis. Details about experimental setup and data analysis procedure can be found elsewhere [28,29].

Before performing electrical/dielectric measurements annealed bars were cut into disks. Thin gold electrodes were sputtered as contacts onto both sides of 1 mm thick sample disks using sputter coater SC7620 (Quorum Technologies Ltd., Laughton, East Sussex, UK). Electrical and dielectric properties were attained by measuring complex impedance using an impedance analyzer (Novocontrol Alpha-AN dielectric spectrometer, Novocontrol Technologies GmbH & Co. KG, Montabaur, Germany) over a wide frequency and temperature range, from 0.01 Hz to 1 MHz at temperatures between 303 K and 523 K. The temperature was controlled to ±0.5 K.

## 3. Results and Discussion

The batch and measured compositions of all IBP glasses are shown in Table 1. It can be seen that compositions match reasonably well, with a clear increasing trend for B_2_O_3_, but slightly fluctuating changes for Fe_2_O_3_ and P_2_O_5_.

For a meaningful analysis of the transport properties of glasses, it is important to consider accurate glass composition especially in the case like this, where the glass composition changes in steps of just a few mol.%. An exact glass composition along with the density of glasses (also shown in Table 1) enables precise calculation of charge carrier (polaron) number density which is an important parameter for polaronic transport. Therefore, in our analysis and discussion of electrical parameters we use the exact composition of glasses throughout the entire manuscript.

**Figure 1:**
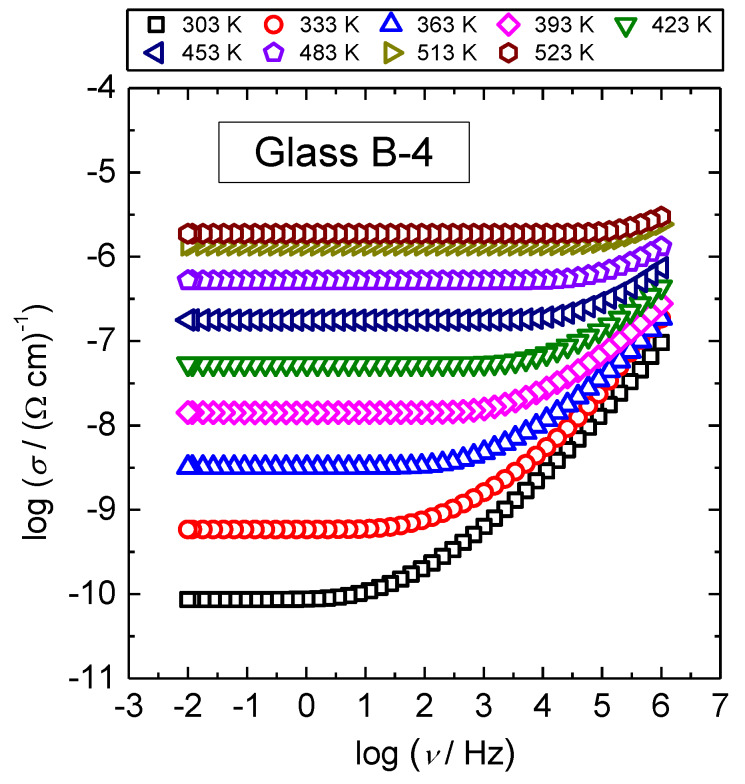
Conductivity spectra for B-4 glass.

Conductivity isotherms of B-4 glass are shown in Figure 1 and are typical spectra for all IBP glasses studied in this work.

Normally, each isotherm exhibits two features, a plateau at lower frequencies that corresponds to the DC conductivity and dispersion at higher ones. The dispersive behavior is more evident at lower temperatures and frequencies, and shifts to higher frequencies with temperature. In Figure 1, the conductivity dispersion for B-4 glass is visible for temperatures up to 483 K. Further, it can be seen that DC conductivity is thermally activated whereas conductivity dispersion shows a weak temperature dependence. In the following, we present in detail the analysis of both quantities in terms of their temperature and compositional dependence.

### 3.1. DC Conductivity

**Figure 2:**
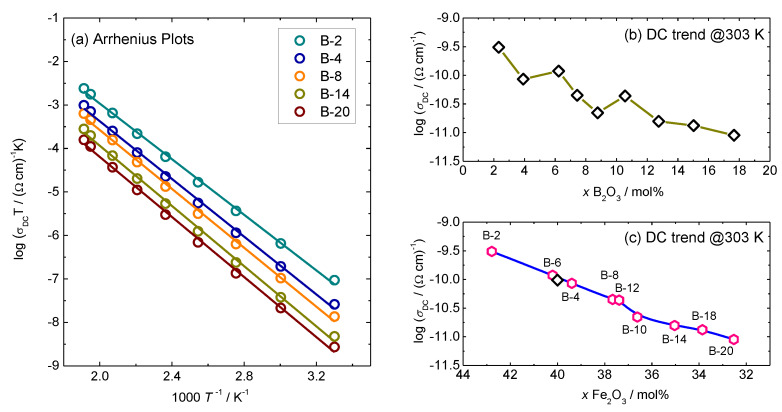
(**a**). Temperature dependence of DC conductivity (log(*σ*_DC_*T*) vs. 1/T) for selected glasses from *x*B_2_O_3_–(100 − *x*)[40Fe_2_O_3_–60P_2_O_5_] series and (**b**,**c**) DC-conductivity at 303 K as a function of B_2_O_3_ and Fe_2_O_3_ content, respectively, for glasses from *x*B_2_O_3_–(100 − *x*)[40Fe_2_O_3_–60P_2_O_5_] series. Solid lines in (**a**) represent the least-square linear fits to experimental data, whereas lines in (**b**,**c**) are drawn as guides for the eye. Black open diamond symbol in (**c**) is the experimental data for 40Fe_2_O_3_-60P_2_O_5_ glass reported in [30].

It is well-known that above room temperature conduction in iron phosphate-based glasses is due to phonon-assisted hopping of polarons between nearest neighboring sites [1,2,3,4,5,6,7] and the DC conductivity is thermally activated with characteristic activation energy. The temperature dependence of DC conductivity, *σ*_DC_, in this temperature range is usually expressed by Austin- Mott’s relation [1,2,5]:

*σ*_DC_ = (*σ*_0_/T) exp((−*E*_DC_)/(k_B_*T*)) (1)

where σ_0_ is the pre-exponential factor, *E*_DC_ is the activation energy for the DC conductivity, k_B_ is the Boltzmann constant and *T* is the temperature (K). In this relation, the pre-exponential factor, σ_0_, contains important parameters for polaronic transport according to the relation:

*σ*_0_ = (*C*(1−*C*)*ν*_ph_*e*^2^)/(R k_B_)) exp(−2αR), (2)

where *C* is the fraction of transition metal (TM) ions in lower oxidation state to total TM ion content and *R* is the average spacing between transition metal ions (*R* = *N*^(−1/3)), *ν*_ph_ is the phonon frequency (≈10^12^–10^13^ Hz), α is the rate of wave function decay, *e* is the electronic charge. The DC-conductivity variation with 1/*T* for selected glasses is shown in Figure 2a.

The activation energy for DC conductivity, *E*_DC_, for each glass is calculated from the slope of log(*σ*_DC_*T*) vs. 1000/*T* and listed in Table 2 along with the values of pre-exponential factor and DC conductivity. The values for DC conductivity at 303 K for all glasses are between 3.08 × 10^−10^ (Ω cm)^−1^ and 8.97 × 10^−12^ (Ω cm)^−1^ and activation energy changes from 0.63 to 0.69 eV which is in good agreement with the values of various iron phosphate-based glasses [6].

**Figure 3:**
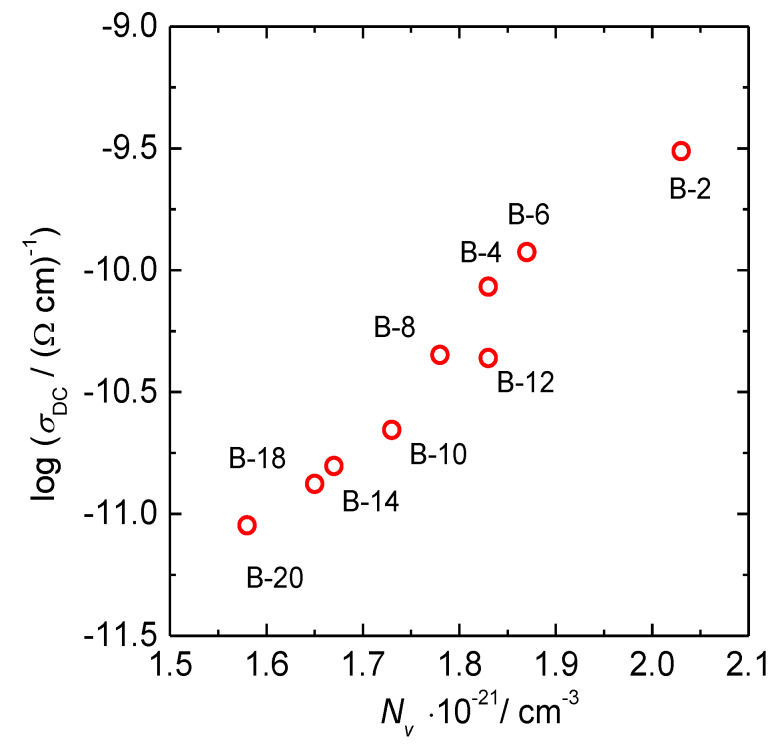
DC conductivity at 303 K versus polaron number density, *N*v, for glasses from *x*B_2_O_3_–(100 − *x*)[40Fe_2_O_3_–60P_2_O_5_] series.

The dependence of DC conductivity of glasses upon B_2_O_3_ and Fe_2_O_3_ content is shown in Figure 2b,c. The DC conductivity decreases perfectly linearly for almost two orders of magnitude with decreasing Fe_2_O_3_ content, whereas it shows a scattered decrease with a simultaneous increase in B_2_O_3_. Observed trends suggest that the dominant parameter for the polaronic transport in these glasses is Fe_2_O_3_ content and not the addition of B_2_O_3_. Interestingly, the DC conductivity of binary 40Fe_2_O_3_-60P_2_O_5_ mol.% glass without B_2_O_3_ which we previously reported [30] lies precisely on the linear dependence of DC conductivity versus Fe_2_O_3_ content of the present glass system, see the black symbol in Figure 2c. This further supports our conclusion that the polaronic transport in these glasses is controlled solely by the iron oxide content and hence, polaron number density. Here, it should be noted that the polaron number density depends not only on the total amount of Fe_2_O_3,_ but also on the fraction of Fe^2+^ and Fe^3+^ ions. For the Fe^2+^/Fe_tot_ ratio ≤ 0.5 the polaron number density is determined by the product of number density of the total iron ions and fraction of ferrous ions, whereas for Fe^2+^/Fe_tot_ ratio > 0.5 it is determined as a product of the number density of the total iron ions and the fraction of ferric ions [3,4,30]. In the previous structural study of this glass system [24], it was shown that there is no significant variation in Fe^2+^/Fe_tot_ ratio since the fraction of ferrous ions for selected glasses changes from 0.16 to 0.22. Therefore, we can approximate Fe^2+^/Fe_tot_ ratio to be constant throughout the entire series, which allows us to assume that the polaron number density is proportional to the overall Fe_2_O_3_ content. The values of polaron number density calculated using the average value of Fe^2+^/Fe_tot_ = 0.19 for all glasses are given in Table 3. For example, the polaron number density, *N*v (polarons), for B-2 glass is calculated by multiplying number density of the total iron ions, N (Fe ions), see Table 3, and 0.19 (10.7 × 10^21^ cm^−3^ × 0.19 = 2.03 × 10^21^ cm^−3^). Indeed, the DC conductivity exhibits a similarly linear increase with increasing polaron number density, see Figure 3.

**Figure 4:**
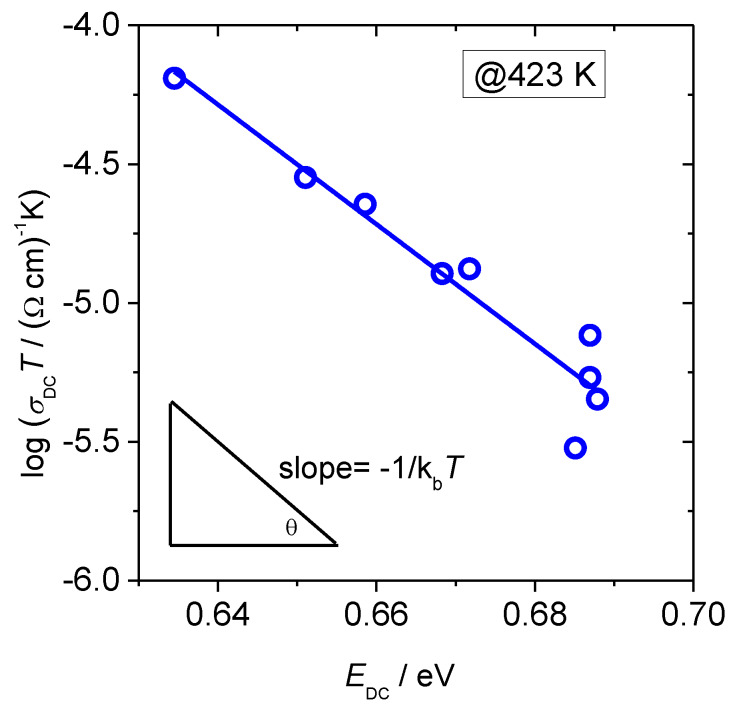
Log(*σ*_DC_*T*) versus *E*_DC_ for studied *x*B_2_O_3_–(100 − *x*)[40Fe_2_O_3_–60P_2_O_5_] glass series. Each point represents an individual glass sample. Solid line represents the least-squares linear fit to experimental data.

Going further in the analysis of the polaronic transport in these glasses we turn our attention to the changes in the pre-exponential factor and parameters it contains, see Table 2. According to Austin–Mott`s theory of small polaron hopping, the conduction process can be characterized by either adiabatic or non-adiabatic hopping. In adiabatic hopping, the electron is at all times relaxed in the potential well of its lattice distortion and hence can respond rapidly to the displacement of the lattice due to the polarization field, while in non-adiabatic hopping the chance of the electron tunneling is rather small [1,2,5]. Hence, in adiabatic hopping conduction, the tunneling term exp(−2*αR*) in Equation (2) is approximately 1, thus *αR* becomes negligible. Although in literature there has been much discussion about whether adiabatic or non-adiabatic hopping picture is valid for oxide glasses at high temperatures [3,4], it was shown from DC conductivity data that non-adiabatic hopping model is more suited to describe polaron transport in iron phosphate-based glasses [27]. In order to check the adiabatic or non-adiabatic nature of the hopping conduction in glasses from this study, we have analyzed the dependence of log(*σ*_DC_*T*) vs. activation energy, *E*_DC_ at a fixed experimental temperature *T* = 423 K, see Figure 4. In such a plot, the slope equals to 1⁄k_B_*T* which enables us to determine the mechanism of the polaron hopping in the following way. If the experimental temperature is approximately equal to the temperature obtained from the slope, the process is adiabatic. On the other hand, if the temperatures differ, the process is non-adiabatic.

The value of the temperature obtained from the slope for glasses in this study is *T* = 253 K which is very different from the chosen experimental temperature, *T* = 423 K This indicates the non-adiabatic hopping of small polarons in these glasses. This result is in accordance with the non-adiabatic hopping mechanism found in similar iron phosphate-based glasses: *x*B_2_O_3_–(40−*x*)Fe_2_O_3_–60P_2_O_5_ (0 ≤ *x* ≤ 20 mol.%) [27], *x*HfO_2_-(40−*x*)Fe_2_O_3_-60P_2_O_5_ (0 ≤ *x* ≤ 8 mol.%), *x*CeO_2_-(40-*x*)Fe_2_O_3_-60P_2_O_5_ (0 ≤ *x* ≤ 8 mol.%) and *x*HfO_2_-(38−*x*)Fe_2_O_3_-2B_2_O_3_-60P_2_O_5_ (2 ≤ *x* ≤ 6 mol.%) [30], CaO–BaO–Fe_2_O_3_–P_2_O_5_ [31], Fe_2_O_3_–Bi_2_O_3_– P_2_O_5_ [32] and Fe_2_O_3_–CaO–P_2_O_5_ [33].

Going back to the influence of B_2_O_3_ on the polaronic transport in these glasses, it seems that the DC conductivity is strongly controlled by the content of Fe_2_O_3_ and independent of the B_2_O_3_ addition and the structural changes it introduces. The IR and Mössbauer spectroscopy study [24] showed that boron enters phosphate network as BO_4_ tetrahedral units forming B–O–B and/or B–O–P type bonds rather than B–O–Fe bonds. Consequently, the iron environment in these glasses is not significantly affected by the addition of B_2_O_3_. Both Fe^2+^ and Fe^3+^ ions have distorted octahedral coordination in all glasses similarly as in many other iron phosphate systems [9,17]. In addition, the O/P ratio for these glasses changes from 3.65 to 4.01, see Table 1, which suggests the presence of pyrophosphate (Q^1^) and ortophosphate (Q^0^) units in phosphate network with a slight tendency for depolymerization with increasing B_2_O_3_ content. The fact that the structural environment of iron ions remains unaffected by the introduction of borate units in the phosphate network can explain why the DC conductivity is not directly dependent on B_2_O_3_ addition.

### 3.2. Frequency-Dependent Conductivity and Scaling Properties

While the analysis of DC conductivity and its temperature dependence gives important information on the long-range transport of charge carriers in glasses, features of the frequency-dependent conductivity provide insights into the processes of their localized motions. A relatively simple, but very helpful means of an analysis of the conductivity spectra over a wide range of frequency and temperatures is based on the application of various scaling procedures. In this respect, one of the simplest and widely used scaling procedures is Summerfield scaling [34,35] which uses experimentally easily assessable parameter—DC conductivity as a scaling factor. The Summerfield scaling is expressed by the relation: *(σ(ν,T)/σ*_DC_*(T)) = F(ν/Tσ*_DC_*(T))* and it can be understood as a mobility scaling; it is indicative that the role of temperature is to only speed up the charge carrier dynamics with increasing temperature without changing the conduction mechanism.

**Figure 5:**
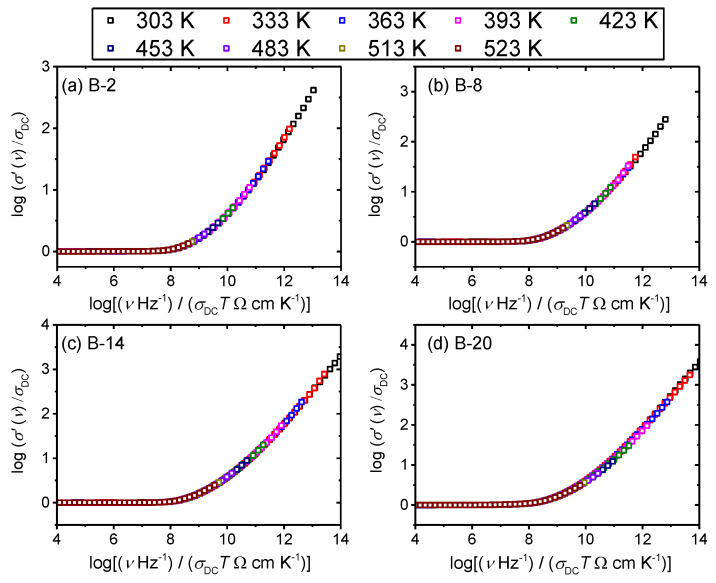
Conductivity spectra scaled according to the Summerfield scaling procedure for (**a**) B-2, (**b**) B-8, (**c**) B-14 and (**d**) B-20 glasses.

For all glasses in this study, Summerfield scaling procedure yields a perfect master-curve demonstrating that the time–temperature superposition (TTS) is valid and that the conductivity mechanism is temperature-invariant, as shown for B-2, B-8, B-14 and B-20 glasses in Figure 5. This is in line with the scaling properties of various oxide glasses that exhibit ionic [36,37] or polaronic conductivity [30,37]. Interestingly, our previous study on *x*B_2_O_3_–(40–*x*)–Fe_2_O_3_–60P_2_O_5_ (*x* = 0–20, mol.%) system showed that glass with 2 mol.% of B_2_O_3_ fails to produce a master-curve when Summerfield scaling procedure is applied [27]. The cause of this deviation was found to be related to the influence of structure on the polaronic conductivity, i.e., local structural disorder induced by the addition of a small amount of B_2_O_3_ which allows changes of the number density of polarons and/or their pathways with temperature.

**Figure 6:**
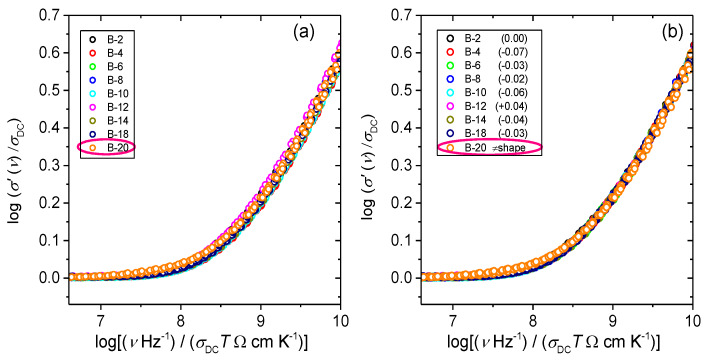
(**a**) Construction of super-master curve of the conductivity isotherms using the Summerfield scaling procedure for *x*B_2_O_3_–(100 − *x*)[40Fe_2_O_3_–60P_2_O_5_] glass series and (**b**) individual conductivity master-curves shifted along the *x*-axis.

In the glass system studied in this work, we have not observed the same effect and glasses with small amounts of B_2_O_3_ obey Summerfield scaling (cf. with Figure 5 for B-2 glass). This result can be related to the fact that within this glass series, B_2_O_3_ is added at the expense of both Fe_2_O_3_ and P_2_O_5_ which has a different influence on the cross-linking of the iron phosphate network. Previous structural study of these two glass systems [24] shows that a gradual substitution of Fe_2_O_3_ and P_2_O_5_ by B_2_O_3_ weakens the network structure as evidenced by the decrease in *T_g_*, whereas an opposite trend is observed for glasses where only Fe_2_O_3_ was replaced by B_2_O_3_. For this reason, it seems likely that at low amounts of B_2_O_3_, small changes in the strength and rigidity of the iron phosphate network affect the mechanism of polaronic transport, i.e., by enabling or disabling the temperature dependence of polaron number density and/or conduction pathways. Furthermore, it is interesting to explore the influence of the glass composition and structure on the conductivity dispersion by applying super-scaling in which all individual master-curves within the glass series are superimposed onto each other. Figure 6a,b shows the result of such super-scaling for all investigated glasses. As can be seen from the Figure 6a individual master-curves do not accurately overlap and a super-master curve could not be obtained. There are two possible reasons for such a result: either the shape of the conductivity dispersion changes or/and individual master curves get shifted along the *x*-axis with the changes in the glass composition.

Intending to detect possible changes in the shape of the conductivity master curves, we have shifted the individual master curves along the *x*-axis in an attempt to produce a super-master curve, see Figure 6b. In the shifting procedure, the master-curve of B-2 glass was taken as a reference curve. All master-curves except that for B-20 glass perfectly overlap when shifted which suggests that the shape of their conductivity dispersion is the same. On the other hand, the master-curve for B-20 glass exhibits evidently different shape of the frequency-dependent conductivity, thus failing to superimpose onto the super-master curve of glasses from B-2 to B-18. Although it is hard to argue about the origin(s) of different shape of the conductivity dispersion for B-20 glass without more detailed structural characterization of the whole glass series and investigation of the glasses containing higher B_2_O_3_ amounts, it is likely related to the structural features of the glass at highest B_2_O_3_ content within this series. Possibly, the extensive inclusion of BO_4_ tetrahedra and formation of B-O-P and B-O-B bonds in this glass influences the local dynamics of polarons.

The values of the shift needed to produce a super-master curve for B-2 to B-18 glasses are given in the legend of Figure 6b. Considering the magnitude and direction of the shift, it can be seen that values are very small and they do not imply any trend with the change of glass composition. The shift in conductivity super-master plot has been reported for various series of ionically conducting oxide glasses where it has been correlated with the alkali oxide content and hence number density of mobile ions [38,39] as well as changes in the typical length of the hop of the ions with their number density [40]. Recently, we have shown that the Summerfield master-curves of polaronically conducting iron phosphate glasses of similar composition and structure, but widely ranging fraction of ferrous ions (0.23 ≤ Fe^2+^/Fe_tot_ ≤ 0.58) also exhibit a shift on the *x*-axis upon super-scaling [30]. Our analysis revealed that the observed shift can be correlated to the product of polaron number density, *N_v_* and the spatial extent of localized motions of polarons, <r^2^_LOC_(∞)>^1/2^. Therefore, it is further of interest to consider the characteristic spatial extent of localized hopping of polarons which can be assessed from the scaling property of the permittivity spectra.

### 3.3. Scaling of Permittivity Spectra and Length Scales of Polaronic Transport

**Figure 7:**
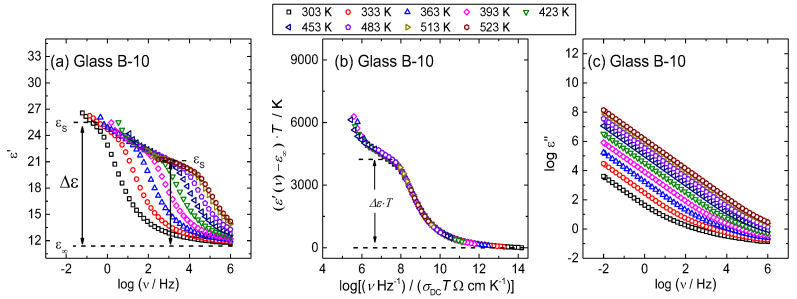
(**a**) Real permittivity spectra at different temperatures, (**b**) their scaled representation obtained using Summerfield scaling procedure and (**c**) imaginary permittivity spectra at different temperatures for glass B-10.

The spectra of the real part of permittivity, *ε*′ (*ν*), at different temperatures for B-10 glass is shown in Figure 7a as a representative for all other glasses in this study. At each temperature, permittivity spectrum exhibits a typical frequency-dependence; at higher frequencies, the real part of the permittivity tends to a constant value, *ε*′_∞_, which results from rapid polarization processes occurring in the glasses under an applied field whereas, with decreasing frequency, it increases and approaches a limiting low frequency plateau denoted as the low-frequency static permittivity, *ε*′_s_. The static permittivity is associated with the polarization effects of the polarons with respect to the immobile glass matrix and determines the difference Δε = *ε*′_s_−ε′_∞_ called dielectric strength of relaxation [40,41].

Here it should be noted that permittivity spectra of polaronic glasses exhibit a well-defined low frequency plateau, as shown in Figure 7a, which is not the case for most ionically conducting glasses since in their spectra effects of electrode polarization dominate at low frequency, hence, masking the permittivity plateau. In contrast, polaronic glasses exhibit a small increase of the real part of permittivity below the point corresponding to static permittivity due to a negligible electrode polarization and surface effects. The imaginary part of permittivity, ε″, increases linearly with decreasing frequency in a log–log plot, see Figure 7c, due to the contribution of DC conduction [42].

**Figure 8:**
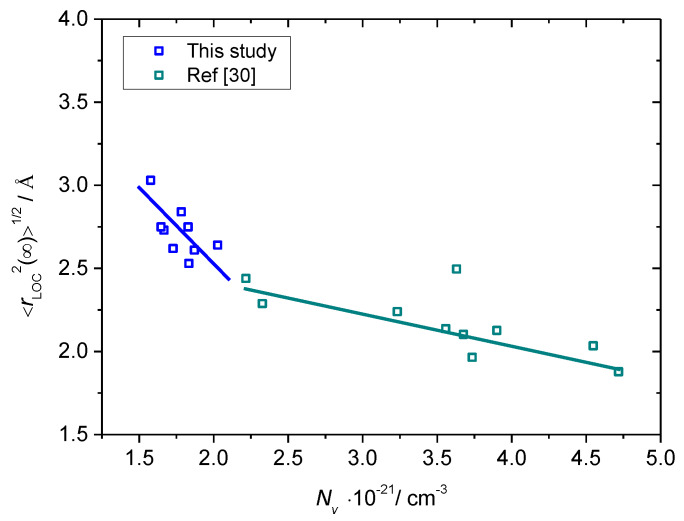
Spatial extent of localized motions of polarons, < *r*_LOC_^2^(∞)>^1/2^, as a function of the number density of polarons for *x*B_2_O_3_–(100 − *x*)[40Fe_2_O_3_–60P_2_O_5_] glasses from this study and HfO_2_-Fe_2_O_3_-P_2_O_5_, CeO_2_-Fe_2_O_3_-P_2_O_5_ and HfO_2_-Fe_2_O_3_-B_2_O_3_-P_2_O_5_ glasses from Ref. [30]. Lines are drawn as guides for the eye.

Since permittivity and conductivity are directly related quantities, the scaling properties of conductivities are mirrored in the scaling properties of permittivity. The Summerfield scaling procedure for the permittivity spectra includes rescaling of the real part of permittivity by subtracting ε′_∞_ and multiplying by *T*, while the *x*-axis is scaled by the product *(Tσ*_DC_*(T))*. Expectedly, from the results of conductivity scaling, the Summerfield scaling procedure yields a perfect permittivity master curve for all glasses in this study. The representative plot of permittivity master-curve is shown for B-10 glass in Figure 7b. For each glass, the scaling of the permittivity spectra yields a parameter Δ*ε* *T* which can be related to the typical spatial extent of localized motions of polarons, <r^2^_LOC_ (∞)>^1/2^. via relation [30,43]:

< *r*_LOC_^2^(∞)>^1/2^ = 6k_B_*ε_0_*Δ*ε T*/*N*_v_q^2^ (3)

where k_B_ is Boltzmann’s constant, *ε_0_* is the permittivity of free space and *N*_v_ is the number density of polarons. For each glass, the parameter Δ*εT* is determined from the onset of the static permittivity plateau in permittivity master-curve as shown graphically for glass B-10 in Figure 7b. The obtained values of the extent of the localized motions of polarons are given in Table 3 and shown in Figure 8.

As can be seen from Table 3 and Figure 8, the value of < *r*_LOC_^2^(∞)>^1/2^ increases from 2.53 Å to 3.03 Å as the polaron number density, *N*_v_, decreases from 2.03 × 10^21^ cm^−3^ to 1.58 × 10^21^ cm^−3^ for B-20 glass. Such a trend corroborates our previous findings [30] that the spatial extent of the localized polaron hopping decreases with increasing polaron number density and represents a realistic estimate of the extent of polaron cavity. Indeed, from Table 3 it is visible that the magnitude of the <*r*_LOC_^2^(∞)>^1/2^ values is close to that of polaron radius, *r_p_*, calculated from the equation proposed by Bogomolov and Mirilin: *r*_p_ = (1/2) (π/6*N*)^1/3^ where *N* is the total number of iron ions [44,45]. Also, it should be noted that the values of < *r*_LOC_^2^(∞)>^1/2^ for these glasses are higher than those obtained for glasses from *x*HfO_2_-(40−*x*)Fe_2_O_3_-60P_2_O_5_, 0 ≤ *x* ≤ 8 mol.%, *x*CeO_2_-(40−*x*)Fe_2_O_3_-60P_2_O_5_, 0 ≤ *x* ≤ 8 mol.% and *x*HfO_2_-(38−x)Fe_2_O_3_-2B_2_O_3_-60P_2_O_5_ 2 ≤ *x* ≤ 6 mol.% series [30]. This result is in line with their lower values of polaron number density, compare Table 3 and Table 3 in [30]. However, it is interesting to see that the decrease of < *r*_LOC_^2^(∞)>^1/2^ with *N*v for the present glass system is characterized by a larger slope than that of HfO_2_-Fe_2_O_3_-P_2_O_5_, CeO_2_-Fe_2_O_3_-P_2_O_5_ and HfO_2_-Fe_2_O_3_-B_2_O_3_-P_2_O_5_ glasses [30], see Figure 8. This observation can be related either to the influence of borate units on the formation of polarons or to the inherent property of the polaronic transport in iron phosphate glasses with low *N*_v_. In order to ascertain the actual cause of this behavior, more investigations on the iron phosphate glasses with a similar range of polaron number density but free of B_2_O_3_ are required. These studies are underway.

**Figure 9:**
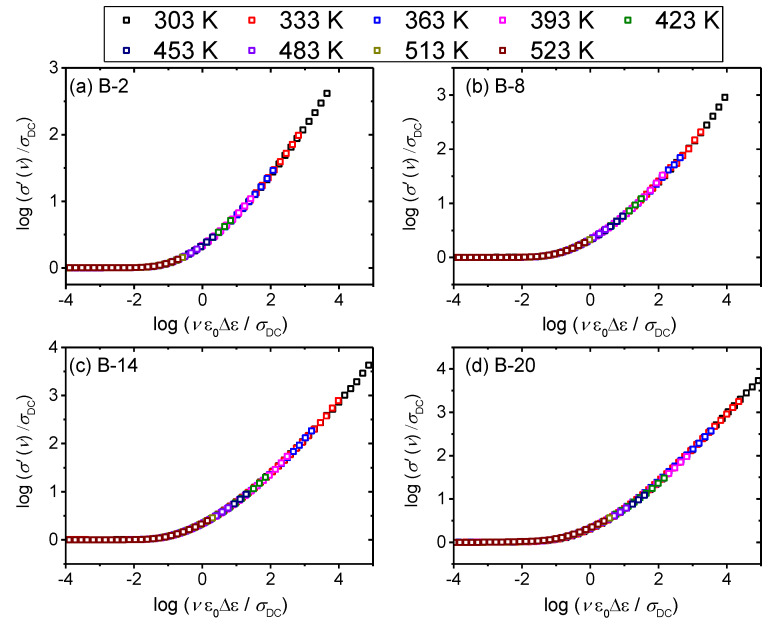
Conductivity spectra scaled according to the Sidebottom scaling procedure for (**a**) B-2, (**b**) B-8, (**c**) B-14 and (**d**) B-20 glasses.

Further, stimulated by the fact that all glasses in our study exhibit a well-defined static permittivity plateau which enables us to precisely determine dielectric strength, Δε, we apply an alternative scaling procedure for conductivity spectra proposed by Sidebottom which is expressed by the form: *(σ(ν,T)/σ*_DC_*(T)) = F(ν*ε_0_Δε*/σ*_DC_*(T))* [40]. The Sidebottom scaling procedure considers the simultaneous change in the typical hopping distance of the charge carriers with changes of their number density and it is considered as truly universal, since it applies whenever scaling is possible at all, i.e., when the shape of the conductivity dispersion does not change with temperature [45]. Figure 9. shows that Sidebottom scaling procedure produces conductivity master-curve for B-2, B-8, B-14 and B-20 glasses and the same result was obtained for all other glasses in this study.

**Figure 10:**
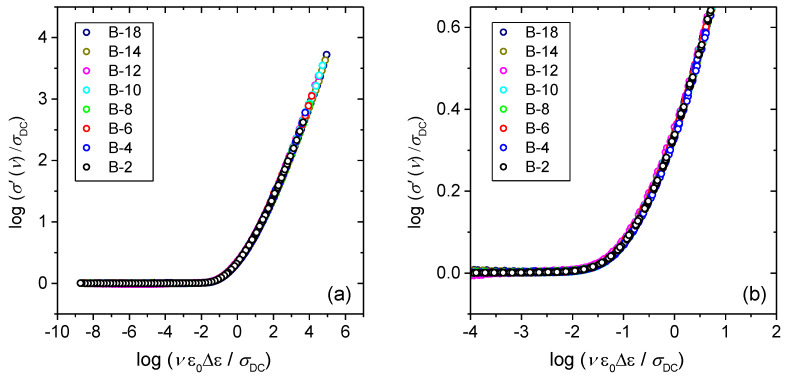
(**a**) Construction of super-master curve of the conductivity isotherms using Sidebottom scaling procedure for B-2 to B-18 glasses and (**b**) zoomed area to underline the observed overlap. B-20 glass is not plotted due to different shape of the conductivity dispersion.

Finally, we show that Sidebottom scaling yields a perfect conductivity super-master curve for glasses from B-2 to B-18, see Figure 10. Having in mind that the Summerfield scaling failed to do so, this result implies that the lengths of a polaron hop change with composition in these glasses.

## 4. Conclusions

The analysis of electrical properties of *x*B_2_O_3_–(100 − *x*)[40Fe_2_O_3_–60P_2_O_5_], *x* = 2–20 mol.% nominal composition glasses in a wide range of temperatures and frequencies reveal that the DC conductivity depends strongly on the polaron number density determined by the overall amount of Fe_2_O_3_. The polaronic transport in all glasses is non-adiabatic. The addition of B_2_O_3_ is not found to be the factor that influences long-range DC conductivity in this glass system, however, it seems that it affects the short-range dynamics of polarons as evidenced in the features of the frequency-dependent conductivity. The consideration of the scaling features of conductivity spectra shows that all glasses obey Summerfield and Sidebottom scaling procedures indicating the validity of the time–temperature superposition principle. Further, the superposition of individual master curves onto each other in an attempt to produce a super-master curve revealed two important results. First, the shape of the conductivity dispersion is the same for glasses with up to 15.0 mol.% B_2_O_3_ but differs for glass with 17.7 mol.% B_2_O_3_. A different shape of the frequency-dependent conductivity of the latter glass is most probably related to the structural features of the glass at high B_2_O_3_ content. Second, while the Summerfield scaling procedure appears to fail, Sidebottom scaling yields a super-master curve for glasses with up to 15.0 mol.% B_2_O_3_, which indicates that while the time–temperature superposition principle is fulfilled, polaronic hopping lengths also change with changing polaron number density in these glasses. The calculated spatial extent of localized motions of polarons, assessed from scaled permittivity spectra, for all glasses are close in values to their polaronic radii. Interestingly, the dependence of the values of the spatial extent of localized polaron motions on the polaron number density complements well the dependence previously found for iron phosphate glasses containing HfO_2_ and CeO_2_ with larger polaron number density. However, this dependence for glasses from this study is characterized by a larger slope which could also be related to the influence of B_2_O_3_ on glass structure and formation of polarons.

## Figures and Tables

**Table 1:** Nominal batch and measured compositions (in mol.%) for studied *x*B_2_O_3_–(100 − *x*)[40Fe_2_O_3_–60P_2_O_5_] glasses.

Glass	Batch Composition (mol.%)	Measured Composition ^a^ (mol.%)	Molar O/P Ratio	Density ^b^ (g cm^−3^)	Fe^2+^/Fe_tot_ Ratio ^b^
B-2	2B_2_O_3_-39.2Fe_2_O_3_-58.8P_2_O_5_	2.3B_2_O_3_-42.8Fe_2_O_3_-54.9P_2_O_5_	3.73	3.06	0.22
B-4	4B_2_O_3_-38.4Fe_2_O_3_-57.6P_2_O_5_	3.9B_2_O_3_-39.4Fe_2_O_3_-56.7P_2_O_5_	3.65	2.97	nm
B-6	6B_2_O_3_-37.6Fe_2_O_3_-56.4P_2_O_5_	6.2B_2_O_3_-40.2Fe_2_O_3_-53.6P_2_O_5_	3.80	2.94	nm
B-8	8B_2_O_3_-36.8Fe_2_O_3_-55.2P_2_O_5_	7.4B_2_O_3_-37.7Fe_2_O_3_-54.9P_2_O_5_	3.73	2.95	0.16
B-10	10B_2_O_3_-36.0Fe_2_O_3_-54P_2_O_5_	8.8B_2_O_3_-36.6Fe_2_O_3_-54.6P_2_O_5_	3.75	2.93	nm
B-12	12B_2_O_3_-35.2Fe_2_O_3_-52.8P_2_O_5_	10.6B_2_O_3_-37.4Fe_2_O_3_-52.1P_2_O_5_	3.88	3.01	nm
B-14	14B_2_O_3_-34.4Fe_2_O_3_-51.6P_2_O_5_	12.7B_2_O_3_-35.0Fe_2_O_3_-52.2P_2_O_5_	3.87	2.89	nm
B-18	18B_2_O_3_-32.8Fe_2_O_3_-49.2P_2_O_5_	15.0B_2_O_3_-33.9Fe_2_O_3_-51.1P_2_O_5_	3.94	2.91	nm
B-20	20B_2_O_3_-32Fe_2_O_3_-48P_2_O_5_	17.7B_2_O_3_-32.5Fe_2_O_3_-49.8P_2_O_5_	4.01	2.86	nm

^a^ composition determined by particle induced gamma-ray emission (PIGE) technique; ^b^ from ref [24], nm—not measured.

**Table 2:** DC conductivity, *σ*_DC_, activation energy, *E*_DC_ and pre-exponential factor, *σ*_0_, for all studied glasses.

Glass	*σ*_DC_^a^/(Ω cm)^−1^ ± 0.5%	*E*_DC_/eV ± 0.5%	log (*σ*_0_/(Ω cm)^−1^ K) ± 0.5%
B-2	3.08 × 10^−10^	0.63	3.43
B-4	8.56 × 10^−11^	0.66	3.26
B-6	1.19 × 10^−10^	0.65	3.27
B-8	4.49 × 10^−11^	0.67	3.19
B-10	2.21 × 10^−11^	0.69	3.14
B-12	4.36 × 10^−11^	0.67	3.13
B-14	1.57 × 10^−11^	0.69	2.99
B-18	1.33 × 10^−11^	0.69	2.92
B-20	8.97 × 10^−12^	0.69	2.71

^a^ values at 303 K.

**Table 3:** Summary of quantities and parameters of polaronic transport for glasses from *x*B_2_O_3_–(100 − *x*)[40Fe_2_O_3_–60P_2_O_5_] series.

Glass	*N* (Fe ions) × 10^−21^/cm^−3^	*N*_v_ (Polarons) × 10^−21^/cm^−3^	*R* = *N*^−1/3^/Å	*r*_p_/Å	(Δ*ε∙T*)/K	<*r*_LOC_^2^(∞)>^1/2^ /Å
B-2	10.7	2.03	4.54	1.93	4937	2.64
B-4	9.65	1.83	4.70	1.89	4098	2.53
B-6	9.84	1.87	4.67	1.88	4476	2.61
B-8	9.35	1.78	4.75	1.91	4767	2.84
B-10	9.09	1.73	4.79	1.93	4165	2.62
B-12	9.62	1.83	4.70	1.89	4828	2.75
B-14	8.77	1.67	4.85	1.95	4338	2.73
B-18	8.67	1.65	4.87	1.96	4365	2.75
B-20	8.30	1.58	4.94	1.99	5060	3.03
